# Complete genome sequence of a Megalocytivirus (family Iridoviridae) associated with turbot mortality in China

**DOI:** 10.1186/1743-422X-7-159

**Published:** 2010-07-15

**Authors:** Cheng-Yin Shi, Kun-Tong Jia, Bing Yang, Jie Huang

**Affiliations:** 1 Key Laboratory for Sustainable Utilization of Marine Fisheries Resource, Ministry of Agriculture; Yellow Sea Fisheries Research Institute, Qingdao 266071, China; 2 Key Laboratory of Mariculture, Ministry of Education; Ocean University of China, Qingdao 266003, China

## Abstract

**Background:**

Turbot reddish body iridovirus (TRBIV) causes serious systemic diseases with high mortality in the cultured turbot, *Scophthalmus maximus*. We here sequenced and analyzed the complete genome of TRBIV, which was identified in Shandong province, China.

**Results:**

The genome of TRBIV is a linear double-stranded DNA of 110,104 base pairs, comprising 55% G + C. Total 115 open reading frames were identified, encoding polypeptides ranging from 40 to 1168 amino acids. Amino acid sequences analysis revealed that 39 of the 115 potential gene products of TRBIV show significant homology to other iridovirus proteins. Phylogenetic analysis of conserved genes indicated that TRBIV is closely related to infectious spleen and kidney necrosis virus (ISKNV), rock bream iridovirus (RBIV), orange-spotted grouper iridovirus (OSGIV), and large yellow croaker iridovirus (LYCIV). The results indicated that TRBIV belongs to the genus *Megalocytivirus *(family Iridoviridae).

**Conclusions:**

The determination of the genome of TRBIV will provide useful information for comparative study of Megalocytivirus and developing strategies to control outbreaks of TRBIV-induced disease.

## Background

Iridoviruses are large, icosahedral, double-stranded DNA (dsDNA) viruses that can infect invertebrates and poikilothermic vertebrates, including insects, fish, amphibians, and reptiles. The viral genomes are both circularly permuted and terminally redundant, which is a unique feature among eukaryotic virus genomes [1-5]. Additionally, iridoviruses infect vertebrates have highly methylated genomes [2,6,7]. Based on particle size, host range, DNA cross-hybridization, the presence of a methyltransferase, and the major capsid protein (MCP) sequence, Iridoviruses are classified into five genera: *Iridovirus*, *Chloriridovirus*, *Ranavirus*, *Lymphocystivirus*, and *Megalocytivirus*. Currently, the entire genomes of more than ten iridoviruses have been completely sequenced. These viruses include lymphocystis disease virus 1 (LCDV-1) and LCDV-China (LCDV-C) of the genus *Lymphocystivirus*; tiger frog virus (TFV), frog virus 3 (FV-3), *Ambystoma tigrinum *virus (ATV), and Singapore grouper iridovirus (SGIV) of the genus *Ranavirus*; infectious spleen and kidney necrosis virus (ISKNV), orange-spotted grouper iridovirus (OSGIV), rock bream iridovirus (RBIV), large yellow croaker iridovirus (LYCIV), red sea bream iridovirus (RSIV) of the genus *Megalocytivirus*; IIV-3 (or Invertebrate iridescent virus 3) of the genus *Chloriridovirus*; and IIV-6 (or Chilo iridescent virus) of the genus *Iridovirus *[6,8-17]. The genomes of these iridoviruses range in size from 105,057 bp (TFV) to 212,482 bp (IIV-6), encoding from 96 to 234 potential open reading frames (ORFs), with G+C contents ranging from 27 to 55%, and complex repeat sequences are very common in these genomes. The genomes exhibit little to no colinearity among genera.

Turbot, *Scophthalmus maximus*, is an important aquaculture species in coastal areas of northern China. The annual production value of farmed turbot in China achieved US$400 million in 2006. Recently, more and more epizootic diseases of farmed turbot in China occurred because of high density stocking and improper management. In 2004, a fish disease causing high mortality and severe damage to turbot cultures was reported in China [18]. The histopathology of the viral infection was characterized by cell hypertrophy in the spleen, kidney, cranial connective tissue, and endocardium. The causative agent was confirmed to be an iridovirus-like virus based on microscopic examination and transmission electron microscopy (TEM). The virus was then classified as an iridovirus and named as turbot reddish body iridovirus (TRBIV) [18]. As the disease was important to turbot culture, we have analyzed and molecularly characterized the complete genome of TRBIV. We also performed phylogenetic analysis of selected TRBIV proteins compared with those of other iridoviruses and discussed the taxonomic position of TRBIV. The determination of whole genome of TRBIV will provide useful information for comparative study of Megalocytivirus and developing strategies to control outbreaks of TRBIV-induced disease.

## Results and discussion

### Determination of the viral genomic DNA sequence

PCR was performed using primers - MCP-irido5: 5' GGAAGCTTCAAGTGAGGAGCG TGA 3' and MCP-irido6: 5' GGGAATTCACAGGATAGGGAAGCC 3', which were designed based on the MCP of SBIV by Sudthongkong et al. [19] has and have been used to amplify the C terminal of coding region of SBIV, RSIV, GSDIV, ALIV and DGIV to detect diseased turbot. Sequencing of the PCR products revealed that the region of TRBIV showed a high degree of sequence identity to SBIV (98%), RSIV (98%), GSDIV (98%), ALIV (98%), DGIV (98%) and RBIV (97%), the identities of the amino acid sequence were also higher than those of the other iridoviruses, such as CIV, MIV, TFV, FV-3, LCDV, LCDV-1, ATV, GIV, and SGIV. Subsequently, with the completion of the sequencing of the MCP and ATPase of TRBIV, the sequence analysis results indicated that TRBIV was much more closely related to RSIV, ISKNV, RBIV, OSGIV and LYCIV than to other iridoviruses. We then designed a primer pairs based on the conserved genes of ISKNV, OSGIV, and RBIV, to amplify the TRBIV genome. The amplified PCR products were approximately 2,000 ~ 6,000 bp in length. The initial PCR products were designed to have at least 200 bp of overlapping sequence. The PCR products were cloned into vector pMD18-T and sequenced in both directions with the universal forward and reverse M13 primers using an ABI PRISM 3700 automated DNA sequencer. Finally, about 6× coverage of the TRBIV genome sequence was accomplished.

The TRBIV genome contained a double-stranded DNA consisting of 110,104 bp, with a G + C content of 55%. Among the sequenced Megalocytivirus, the size of TRBIV genome was the smallest, comparing with that of ISKNV (111,362 bp), LYCIV (111,760 bp), RBIV (112,080 bp), RSIV (112,414 bp) and OSGIV (112,636 bp) [13-15]. Comparing with other sequenced iridoviruses, the size of TRBIV genome was higher than that of LCDV-1 (102,653 bp), TFV (105,057 bp), FV-3 (105,903 bp), ATV (106,332 bp), but smaller than that of SGIV (140,131 bp), LCDV-C (186,250 bp), and much smaller than that of CIV (212,482 bp) [6,8-12,17]. The G + C content of the TRBIV genome was similar to those of ISKNV (54.8%), OSGIV (54%), RBIV (53%), FV-3 (55%), TFV (55%), ATV (54%), and SGIV (48.64%), but much higher than that of LCDV-1 (29.1%), LCDV-C (27.25%) and CIV (28.6%) (Table 1).

**Table 1:** Summary of genomic information for 13 sequenced iridoviruses

Virus	Genus	Genome size (bp)	G+C content (%)	No. of ORFs	ORF size (aa)	Year determined	**GenBank Accession no**.
TRBIV	*Megalocytivirus*	110104	55.0	115	40 ~ 1168	2008	GQ273492
ISKNV	*Megalocytivirus*	111362	54.8	124	40 ~ 1208	2001	AF371960
OSGIV	*Megalocytivirus*	112636	54.0	121	40 ~ 1168	2004	AY894343
RBIV	*Megalocytivirus*	112080	53.0	118	50 ~ 1253	2004	AY532606
FV3	*Ranavirus*	105903	55.0	98	50 ~ 1293	2004	AY548484
TFV	*Ranavirus*	105057	55.0	106	40 ~ 1294	2002	AF389451
ATV	*Ranavirus*	106332	54.0	96	32 ~ 1294	2003	AY150217
GIV	*Ranavirus*	139763	45.0	120	62 ~ 1268	2005	AY666015
SGIV	*Ranavirus*	140131	48.6	162	41 ~ 1268	2004	AY521625
LCDV-1	*Lymphocystivirus*	102653	29.1	195	40 ~ 1199	1997	L63545
LCDV-C	*Lymphocystivirus*	186247	27.3	240	40 ~ 1193	2004	AY380826
CIV	*Iridovirus*	212482	28.6	468	40 ~ 2432	2001	AF303741
MIV	*Chloriridovirus*	190132	47.9	126	60 ~ 1377	2006	DQ643392

The TRBIV genome contained numerous short direct, inverted, and palindromic repetitive sequences. The highly (direct) repetitive region was identified at position 23,512 to 23,762 bp in the TRBIV genome, which was also found in ISKNV and OSGIV [14,15]. The highly (direct) repetitive region showed highly homologies to the repetitive regions of ISKNV (78%) and OSGIV (78%).

### Coding capacity of TRBIV genome

**Figure 1:**
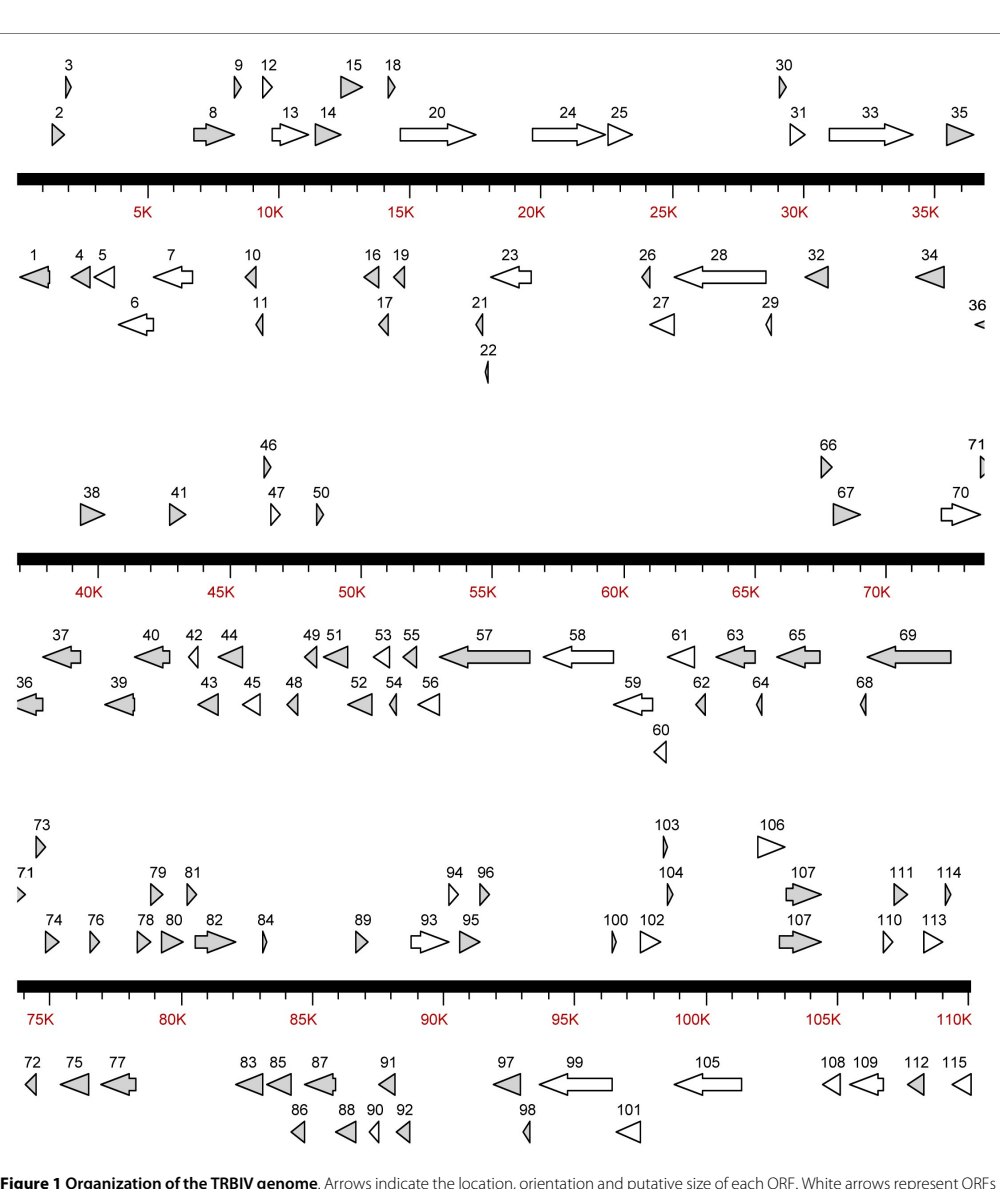
**Organization of the TRBIV genome**. Arrows indicate the location, orientation and putative size of each ORF. White arrows represent ORFs with predicted function similar to other organisms, and black arrows represent ORFs with unknown function.

The prediction of presumptive genes was carried out using the DS GENE 1.5 viral gene prediction program (Accelrys Inc.) and NCBI ORF finder http://www.ncbi.nlm.nih.gov/gorf/gorf.html. A total of 115 presumptive ORFs were identified, encoding proteins ranging from 40 to 1,168 amino acids on the sense (R) and antisense (L) DNA strands (Fig. 1 and Additional file 1). The percent coding density of TRBIV is 92%, which is similar to ISKNV (93%), OSGIV (91%), and RBIV (86%) [13-15]. Of the 115 ORFs, 65 (56.5%) were encoded by ORFs present in the forward orientation and 50 (43.5%) in the reverse. The 115 ORFs contained 26 core genes that can be found in all iridoviral genomes [20].

There are 17 pairs of overlapping ORFs in the TRBIV genome. Eleven of these 17 pairs of ORFs have an overlapping of 1 ~ 7 bp. This is similar to ISKNV (ORF 39R and 40L, 45L and 46L, 80L and 81R, 86L and 87R), RBIV (45L and 46L, 57L and 58L), OSGIV (ORF12L and ORF13L, ORF28R and ORF29L, ORF37L and ORF38R, ORF41R and ORF42L, ORF47L and ORF48L, ORF49R and ORF50R, ORF60L and ORF61L, ORF68L and ORF68R, ORF76R and ORF77R, ORF89R and ORF90L, ORF107RL and ORF108L), ATV (ORF 6R and 6bR, 43R and 43bR, 61R and 61bR), and SGIV (ORF1L and 2R, 7L and 8L, 12L and 13R) [10,11,13-15].

### Comparison to other iridoviruses isolated from fish

TRBIV is similar to three other *Megalocytiviruses *(ISKNV, OSGIV and RBIV) in overall genome structure and exhibited colinearity among them. An analysis of the amino acid sequences deduced from the individual ORFs revealed that 23 of ORFs were the same as the ORFs of ISKNV, OSGIV, RBIV in size and orientation, with homologies ranging from 85% to 99% at the amino acid level. 94, 105 and 111 ORFs of TRBIV had homologies to those of RBIV (58-100%), ISKNV (66-100%) and OSGIV (66-100%) at the amino acid level [13-15]. Comparing TRBIV with ISKNV, 42 ORFs were the same size and the identities ranged from 84% to 99%. Comparing TRBIV and OSGIV, 37 of the 115 ORFs were the same size and their identities varied from 87% to 99%; 35 of the 115 ORFs differed in size with identities raging from 66% to 99%. Comparing TRBIV and RBIV, 30 ORFs were the same size and the identities ranged from 77% to100%.

The complete sequence of TRBIV was compared to all iridovirus sequences available in the data banks. The result showed that the putative gene products of TRBIV shared high homology to the corresponding viral proteins of other iridoviruses (Additional file 1). There are 26 core genes which can be found in all iridovirus genomes, such as Serine/threonine protein kinases, DNA polymerase (DdDP), DNA methyl transferase (DMet), SNF2-like helicase, DNA repair protein (RAD2), large subunits of DNA-dependent RNA polymerase (DdRPI and DdRPII), RNase III (RIII), ATPase and MCP. These genes are involved in virus replication, transcription, modification, and structural composition.

### Sequence similarities to proteins in databases

The deduced gene products of the 115 ORFs were compared to amino acid sequences by the NCBI BLAST program http://www.ncbi.nlm.nih.gov/BLAST/. Thirty-nine ORFs showed significant homology to functionally characterized proteins from other species. These proteins included structural proteins and enzymes involved in virus replication, transcription, protein modification, and virus-host interaction (Additional file 1).

### DNA replication and repair

The TRBIV genome contained several genes with predicted roles in viral DNA replication, modification, and processing, such as DNA polymerase (ORF20R), DNA repair protein RAD2 (ORF27L), cytosine DNA methyl transferase (ORF45L), putative replication factor (ORF56L), SNF2 family helicase (ORF 58L), and D5 family NTPase (ORF99L). These ORFs showed 94 ~ 98% identity to those of ISKNV, OSGIV, and RBIV at the amino acid level.

TRBIV ORF45L encoded a homolog of cytosine DNA methyltransferase. High levels of methylation of cytosine at CpG residues were identified in iridoviruses from vertebrate hosts, such as FV3, LCDV-1, EHNV, ISKNV, TFV and OSGIV [7,15,20-22]. DNA methylation is carried out by a group of enzymes called DNA methyltransferases. These enzymes not only determine the DNA methylation patterns during early development, but are also responsible for copying these patterns to the strands generated from DNA replication. In vertebrate iridoviruses, methyltransferases might play a crucial role in the expression of the viral genome [7].

TRBIV ORF20R encoded a protein showing significant similarity to DNA polymerase. DNA polymerase catalyzes the polymerization of deoxyribonucleotides into a DNA strand. DNA polymerases are best known for their role in DNA replication. Like other DNA polymerases, ORF20R consisted of 3'-5'exonuclease and polymerization motifs located at the N-terminal and C-terminal portions of the protein. These highly conserved motifs are important characteristics of the enzyme [23,24].

### Transcription and nucleotide metabolism

TRBIV encodes at least six enzymes that are involved in transcription and nucleotide metabolism. They are ribonucleotide reductase small subunit (ORF25R), two large subunits of the DNA-dependent RNA polymerase (ORF28L, ORF33R), transcription elongation factor IIS (TFIIS, ORF29L), mRNA capping enzyme (ORF59L), and ribonuclease III (ORF80L). These genes of TRBIV are similar to those found in ISKNV and they showed 95 ~ 96% amino acid sequence identities.

TFIIS is ubiquitous in many organisms and plays an important role in transcript elongation [25,26]. Stage and tissue-specific gene expression is an initial and important step in establishing the fate of each cell during development. Expression of particular genes or gene families could be regulated by tissue-specific S-II at the level of transcriptional elongation [27].

### Host-related functions

TRBIV encodes at least 15 proteins involved in host-related functions. ORF12R, 60L, 61L, 90L and 110R encode proteins of various sizes, which contain a RING-finger-containing ubiquitin ligase domain. They are highly homologous to those of ISKNV (ORF12R, 65L, 66L, 99L, and 119R). The study on Ring finger proteins (RFPs) showed that the four RFPs of ISKNV (ORF12R, 65L, 66L, and 111L) acted as the E3 enzyme in the presence of ubiquitin activating enzyme (E1), ubiquitin, zinc ions, and a specific E2 protein (UbcH5 subfamily). The RING domain of RFP in ISKNV (ORF66L) was proved to be essential for the activity of E3 enzyme by mutational analysis [28].

ORF36L encoded a protein of 449 amino acids that showed homology to ORF37L of LYCIV, containing a biologically active RGD tri-peptide. The RGD-containing proteins have been identified to be involved in virus infection and virus-host interaction by their RGD motif interaction with the host cell surface receptors. The study on 37L of LYCIV suggested that it had an important role in virus infection [29-33].

ORF47R, which showed homology to the ORF48R of ISKNV, showed higher similarity to the vascular endothelial growth factor (VEGF) encoded by *Megalocytivirus *and *Parapoxvirus *than to those encoded by fish and mammals. VEGF functions as a specific mitogen for vascular endothelial cells and as a potent inducer of vascular permeability. The study on ORF48R of ISKNV demonstrated that ISKNV ORF48R functions as a potent growth factor to stimulate angiogenesis and can bind its receptor, FLK-1, to affect zebra fish early embryonic vascular development [34].

In addition, some important putative genes involved in host-related function were found in the TRBIV genome, such as ankyrin repeat motifs (ORF70R, 93R, 109L, 115L), the C-terminal domain (CTD)-like phosphatase (ORF5L), proliferating cell nuclear antigen (ORF9R), Src homology 2 (SH2) domain-containing protein, laminin-type epidermal growth factor (EGF)-like domain (ORF24R), and tumor necrosis factor receptor-associated factor (TRAF, ORF101L).

### Putative membrane-associated proteins

The ORFs of TRBIV were analyzed for the presence of putative transmembrane domains (TMs). One or more putative TMs (1 ~ 11) were found in five ORFs (ORF1L, 7L, 11L, 48L, 84R, 112L), using the computer software TMHMM 2.0 [35]. ORF1L exhibited high homology to the amino acid sequences of ORF1L of ISKNV and OSGIV [14,15]. ORF1L of ISKNV, containing 10 ~ 11 putative transmembrane domains, is a membrane protein and is considered to serve as a model for analyzing the topology and roles of different hydrophobic regions in multi-transmembrane proteins [36].

### Other proteins

TRBIV also encoded proteins homologous to a phosphatase (ORF107R), an ATPase (ORF113R) and a structural protein (MCP, ORF6L). ORF6L encoded a protein of 453 amino acids, which showed similarity to MCP of other iridoviruses (i.e., RSIV, DGIV, RBIV, SBIV, OSGIV, and ISKNV) [14,19].

### Relationship of TRBIV to ISNNV, OSGIV and RBIV

The comparative analysis of the TRBIV, ISKNV, OSGIV and RBIV genomes revealed many homologues (Additional file 1). Many ORFs of the four iridovirus genomes, including the conserved genes and other ORFs of unknown function, were similar in size, structure and composition. Total 94, 105 and 111 ORFs of TRBIV had homologies to those of RBIV (58-100%), ISKNV (66-100%), and OSGIV (66-100%) at the amino acid level [13-15]. Twenty-three ORFs of TRBIV had same size and the identities varied from 85% to 99%.

**Figure 2:**
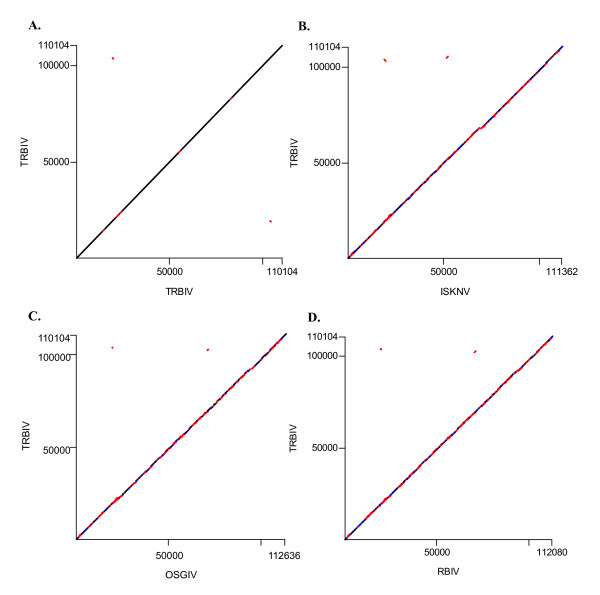
**Dot matrix plots comparing the TRBIV genome (vertical axis) with the ISKNV, OSGIV, RBIV genomes, and itself (horizontal axis)**. The horizontal axes represent (A) the TRBIV genome; (B) the ISKNV genome; (C) the OSGIV genome; and (D) the RBIV genome. The complete genomic sequences were aligned using DS GENE 1.5.

DNA dot matrix analyses of TRBIV genomic DNA with itself, ISKNV, OSGIV and the RBIV genome were performed using DS GENE 1.5 (Accelrys Inc.). The results revealed that the gene order among TRBIV, ISKNV, OSGIV and RBIV was markedly conserved (Fig. 2).

There are many different genes compared with ISKNV, OSGIV and RBIV in the non-coding region and coding region of TRBIV, including gene insertions and deletions (results not shown). Interestingly, there is an obvious gene deletion of about 1070bp compared with ISKNV at position 67468 ~ 67518 of TRBIV, but hardly any difference with OSGIV and RBIV. The differences of the genes among the three viruses reflect their host specificities.

### Relationship of TRBIV to other iridoviruses

Iridovirus sequences available in data banks were compared to the complete sequence of TRBIV. The result showed that the putative gene products of TRBIV shared high homology to the corresponding viral proteins of other iridoviruses (Additional file 1). There were some homologous genes in the ISKNV, OSGIV, RBIV, TFV, FV-3, ATV, SGIV, LCDV-1 and LCDV-C genomes. These included genes for DNA polymerase (DdDP), DNA methyl transferase (DMet), two large subunits of DNA-dependent RNA polymerase (DdRPI and DdRPII), RNase III (RIII), ATPase and MCP, involved in virus replication, transcription, modification, and structural composition.

### Phylogenetic analysis

**Figure 3:**
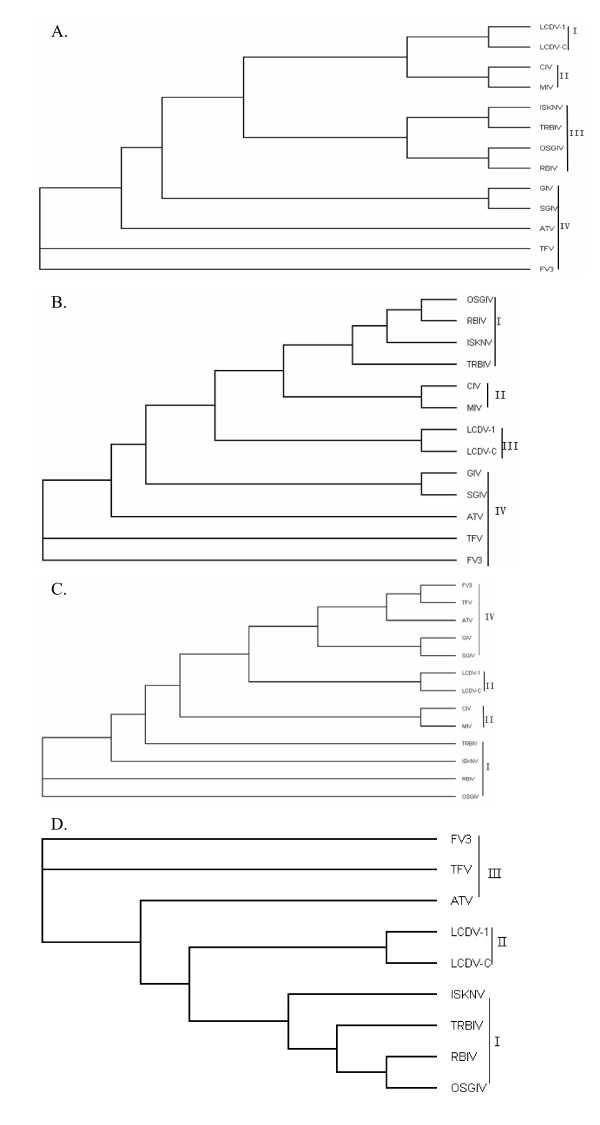
**Phylogenetic relationships of iridoviruses obtained using four protein sequence alignments: (A) major capsid protein, (B) ATPase, (C) DNA polymerase, and (D) cytosine DNA methyl transferase**. The alignments were carried out using Clustal X 1.83 and the neighbor-joining trees obtained using PHYLIP 3.67 are shown with the statistical support indicating the robustness of the inferred branching pattern as assessed using the bootstrap test. GenBank accession numbers: (A) ATV, YP_003785; CIV, NP_149737; MIV, YP_654586; FV3, YP_031669; ISKNV, NP_612228; LCDV-1, NP_044812; LCDV-C, YP_025102; RBIV, AAT71822; SGIV, AAS18087; GIV, AAV91066; TFV, AAL77814; and OSGIV, AAX82316. (B) ATV, AAP33264; CIV, NP_149538; GIV, AAV91100; FV3, YP_031593; OSGIV, AAX82427; ISKNV, AAL98847; LCDV-1, NP_078656; LCDV-C, YP_073620; SGIV, YP_164229; MIV, YP_654660; TFV, AAL77796; and RBIV, AY532606.1. (C) ATV, YP_003817; CIV, AAD48150; FV3, YP_031639; ISKNV, AAL98743; LCDV-1, NP_078724; LCDV-C, YP_073706; RBIV, AAT71835; SGIV, AAS18143; TFV, AAL77804; OSGIV, AAX82331; and MIV, YP_654692. (D) ATV, YP_003792; FV3, YP_031662; ISKNV, AAL98770; OSGIV, AAX82357; LCDV-1, NP_078617; LCDV-C, YP_025103; RBIV, AAT71861; and TFV, NP_572009.

To determine the phylogenetic relationship of TRBIV to other iridoviruses, the amino acid sequences of MCP, ATPase, cytosine DNA methyl transferase and DNA polymerase were used in alignments with other iridoviruses and non-iridoviruses from GenBank. MCP is highly conserved in iridoviruses [37,38]. In the MCP tree, the iridoviruses used in the multiple alignments were subdivided into four groups: group I, *Lymphocystiviruses*, including LCDV-1 and LCDV-C; group II, the insect iridoviruses, including CIV, MIV; group III, *Megalocytiviruses*, including TRBIV, ISKNV, OSGIV, RBIV; and group IV, *Ranaviruses*, including FV3, TFV, ATV, GIV and SGIV (Fig. 3A). The results showed that TRBIV was more closely related to ISKNV, OSGIV and RBIV, than to the *Ranavirus *and *Lymphocystiviruses*. Phylogenetic analyses using the highly conserved full-length protein sequences of the ATPase, cytosine DNA methyl transferase and DNA polymerase from the iridoviruses (Figs. 3B, C, D) also supported the view that TRBIV was more closely related to ISKNV, OSGIV and RBIV.

## Conclusions

In summary, the present study provided the complete genome sequence of TRBIV. The genomic organization, gene content, and amino acid composition of TRBIV are very similar to those of ISKNV, OSGIV and RBIV. Dot plot analyses and phylogenetic trees suggested that TRBIV belongs to the genus *Megalocytivirus *in the family *Iridoviridae*. The detailed viral genome analysis will provide valuable information to serve as the genetic basis for future studies and to control iridovirus disease in cultured fish.

## Methods

### Infected fish

TRBIV used in this study was originally isolated from cultured turbot (*Scophthalmus maximus*) with TRBIV disease from Shandong Province, China, in 2005. These fish had enlarged spleen and kidney cells, as verified by histopathology. The spleens and kidneys were removed from the diseased fish and the viruses were observed by light and electron microscopy. PCR was performed for further confirmation of the disease. These samples were collected and kept at -80°C for future use.

### Virus and viral DNA

Total DNA was extracted from spleen and kidney of diseased turbot using high pure PCR template preparation kit (Roche, USA). The quality and concentration were determined by agarose gel electrophoresis and by a spectrophotometer.

### PCR amplification and sequencing

The complete genomic DNA of TRBIV was sequenced by PCR method. As TRBIV showed high homology to ISKNV, RBIV, and OSGIV, the primers were derived from the DNA sequence of ISKNV, RBIV, and OSGIV in GenBank/EMBL/DDBJ database (AF371960, AY532606, AY894343). Amplified PCR products were about 2000 ~ 6000 bp in length, and the initial PCR products were designed to have at least 200 bp of overlapping sequence. The PCR products were purified using TaKaRa Agarose Gel DNA Purification Kit Ver.2.0 (TaKaRa). The purified PCR products were cloned into vector pMD18-T (TaKaRa), and sequencing was performed using an ABI PRISM 3700 automated DNA sequencer in both directions with the universal forward and reverse M13 primers.

### DNA sequence analysis

Genomic DNA composition, structure, homologous regions and repeated sequences were analyzed using DNASTAR (Lasergene) and OMIGE 2.0 programs. The ORFs and their amino acid sequences were predicted using DS GENE 1.5 (Accelrys Inc.) and NCBI ORF finder http://www.ncbi.nlm.nih.gov/gorf/gorf.html. Protein database searches were conducted using the BLASTP at NCBI Web site http://www.ncbi.nlm.nih.gov/. Comparison of the homologous sequence regions of TRBIV, ISKNV, OSGIV, RBIV, LYCIV and other iridoviruses was performed with BLAST programs. DNA dot matrix plots were obtained using DS GENE 1.5 (Accelrys Inc.). Phylogenetic trees were generated using the PHYLIP package and TreeView. Prediction of transmembrane domains (TMs) was performed using TMHMM 2.0 http://www.cbs.dtu.dk/services/TMHMM-2.0/[35,39].

ORFs were identified by the following criteria: (1) they were not less than 120 bp and (2) they were not located within larger ORFs. In addition, TRBIV ORFs that were likely to be expressed were also identified on the basis of significant identity to known protein sequences within the databases.

### Nucleotide sequence accession number

The complete nucleotide sequence of the TRBIV genome was deposited in GenBank under accession number: GQ273492.

## Competing interests

The authors declare that they have no competing interests.

## Authors' contributions

C-Y S and J H conceived the study. B Y participated in sample collection. C-Y S and K-T J carried out the experiments, data analysis and wrote the manuscript. All authors have read and approved the manuscript.

## Supplementary Material

Additional File 1 **Potential Open Reading Frames of the TRBIV genome and comparative analysis of TRBIV to other iridoviruses**. Click here for file
