# Supplementary material for: Complete genome sequence of a Megalocytivirus (family Iridoviridae) associated with turbot mortality in China
Source: Virol J. 2010 Jul 15;7:159. doi: 10.1186/1743-422X-7-159 (PMC2912838; doi:10.1186/1743-422X-7-159)
Supplement: Additional File 1 — Potential Open Reading Frames of the TRBIV genome and comparative analysis of TRBIV to other iridoviruses. [file 1743-422X-7-159-S1.DOC]

| ORF | Position | Length  (aa) | Conserved domain or signature (CD accession no) | Predicted  Structure  or function | Homologies to ISKNV | | Homologies to OSGIV | | Homologies to RBIV | | Homologies to other iridovirus | | |
| --- | --- | --- | --- | --- | --- | --- | --- | --- | --- | --- | --- | --- | --- |
|  |  |  |  |  | ORF | Identity* | ORF | Identity* | ORF | Identity* | Species | Identity* | Accession no. |
| 1L | 128-1264 | 378 | Transmembrane amino acid transporter protein  （pfam01490） | Transmembrane amino acid transporter protein | 1L | 93 | 1L | 93 | 1L | 93 | LYCIV 94 ABI32390 | | |
| 2R | 1388-1801 | 137 |  |  | 2R | 87 | 2R | 86 |  |  |  | | |
| 3R | 1892-2107 | 71 |  |  |  |  |  |  | 3R | 77 |  | | |
| 4L | 2067-280 | 244 |  |  | 3L | 80 | 4L | 83 | 4L | 83 |  | | |
| 5L | 2970-3716 | 248 | catalytic domain of ctd-like phosphatase  (smart00577) | catalytic domain of ctd-like phosphatase | 5L | 90 | 6L | 93 | 6L | 93 | GIV 37R 33 AAV91058  SGIV 61R 33 AAS18076  LCDV-1 82L 35 NP_078678  TFV 40R 32 ABB92302  FV3 37R 31 YP_031615  LCDV-C 148L 36 YP_073653  ATV 64R 31 YP_003838  CIV 355R 26 NP_149818  MIV 104L 26 YP_654676 | | |
| 6L | 3862-5223 | 453 | Iridovirus major capsid protein (pfam04451) | Iridovirus major capsid protein | 6L | 99 | 7L | 99 | 7L | 99 | Korean flounder iridovirus 99 AAT48721  Korean flounder iridovirus 99 AAT48722  Korean flounder iridovirus 99 AAT48724  Korean flounder iridovirus 99 AAT48726  Olive flounder iridovirus 99 ABY16712  Korean flounder iridovirus 99 AAT48714  Korean flounder iridovirus 99 AAT48723  Olive flounder iridovirus 99 AAT76908  Korean flounder iridovirus 98 AAT48715  Korean flounder iridovirus 98 AAT48725  Korean flounder iridovirus 98 AAT48717  Korean flounder iridovirus 98 AAT48719  Korean flounder iridovirus 98 AAT48716  Murray cod iridovirus 98 AAY18351  DGIV 98 AAY58048  ALIV 98 AAP37442  Korean flounder iridovirus 98 AAT48718  GSDIV 98 AAP74203  SBIV 98 AAP74203  Korean flounder iridovirus 97 AAT48720  DGIV 97 AAP37441  RSIV 97 BAC66968  TBIV 99 BAD12494  LYCIV 97 AAX93516  GSIV 97 ABY27294  LCDV-C 43L 48 YP_025102  LCDV-1 147L 49 NP_044812  EHNV 46 AAO32315  CIV 274L 45 NP_149737  SGIV 72L 44 AAS18087  GIV 45R 44 AAV91066  ATV 14L 46 YP_003785  CIV 274L 45 NP_149737  MIV 14L 44 YP_654586 | | |
| 7L | 5240-6697 | 485 |  | myristylated membrane protein | 7L | 97 | 8L | 97 | 8L | 97 | RSIV 97 BAC66966  King grouper iridovirus 97 ABU68440  RSIV 97 BAC66967  GIV 59L 30 AAV91076  LCDV orf20 31 AAK54492  SGIV 88L 30 AAS18103  TFV 55R 30 ABB92314  FV3 53R 30 ATT09712  MIV 6R 30 ABF82036  LCDV-1 67L 25 NP078665  CIV 118L 25 NP_149581  LCDV-C 148L 36 YP_073653 | | |
| 8R | 6769- 8319 | 516 |  |  | 8R | 85 | 9R | 87 | 9R | 87 | RSIV 87 BAC66966 | | |
| 9R | 8316-8576 | 86 |  |  | 9R | 92 | 10R | 100 | 10R | 100 |  | | |
| 10L | 8735-9127 | 130 |  |  | 10L | 95 | 11L  12L | 97  89 | 11L | 94 |  | | |
| 11L | 9127-9384 | 85 |  |  | 11L | 98 |  |  |  |  |  | | |
| 12R | 9404-9736 | 110 | RING-finger-containing ubiquitin ligase (COG5540) | RING-finger containing  ubiquitin ligase | 12R | 95 | 14R | 95 | 12R | 95 |  | | |
| 13R | 9743-11140 | 465 | Serine/threonine protein  kinases, catalytic domain  (cd00180) | Serine/threonine  protein kinases | 13R | 88 | 15R | 90 | 15R | 90 | LCDV-C 178L 29 YP_073682  SGIV 150R 35 AAS18165  LCDV-143L 25 NP_078729  GIV 100L 35 AAV91110  CIV 98R 31 NP_149561  FV3 57R 30 AAT09717  MIV 98L 30 NP_654670  ATV 47L 30 YP_003820 | | |
| 14R | 11395-12372 | 325 |  |  | 14R | 92 | 16R | 93 | 14R | 93 | *Dwarf gourami* iridovirus 36 AAY58051 | | |
| 15R | 12378-13163 | 261 |  |  | 15R | 92 | 17R | 94 | 15R | 94 |  | | |
| 16L | 13220-13807 | 195 |  |  | 16L | 91 | 18L | 91 | 16L | 91 |  | | |
| 17L | 13822 -14151 | 109 |  |  |  |  | 19L | 86 | 17L | 87 |  | | |
| 18R | 14177 -14413 | 78 |  |  |  |  | 20R | 91 | 18R | 90 |  | | |
| 19L | 14349-14795 | 148 |  |  |  |  | 21L | 84 | 19L | 84 |  | | |
| 20R | 14649-17495 | 948 | DNA polymerase type-B family  DNA directed DNA polymerase (cd00145) | DNA polymerase | 19R | 95 | 22R | 95 | 20R | 95 | RSIV 95 BAA28669  LCDV-C 203L 37 YP_073706  LCDV-1 135R 36 NP_078724  GIV 87R 35 AAV91098  SGIV 128R 35 AAS18143  FV3 60R 35 YP_031639  TFV 63R 35 NP_572000  MIV 120R 30 YP_654692  CIV 37L 29 CAC84133 | | |
| 21L | 17529-17750 | 73 |  |  | 20L | 88 | 23L | 91 | 21L | 91 |  | | |
| 22L | 17847-17969 | 40 |  |  | 21L | 87 | 24L | 92 |  |  |  | | |
| 23L | 18059-19597 | 512 | Appr-1”-p processing  enzyme (smart00506) | putative phosphatase | 22L | 88 | 25L | 96 | 22L | 86 | Sea perch iridovirus ORF-1 99 AAV51312  RSIV 85 AAQ076955  Rock bream iridovirus ORF-1 96 AAN86691  ISKNV 117R 45  OSGIV 113R 44 | | |
| 24R | 19670 -22432 | 920 | Laminin-type epidermal  growth factor-like  domain (cd00055) | Laminin-type epidermal growth factor-like protein | 23R | 66 | 26R | 67 | 23R | 75 | Sea perch iridovirus ORF-2 97 AAV51313  Olive flounder iridovirus 90 AAT76907  LYCIV 72 ABI32391  RSIV 66 AAQ07956 | | |
| 25R | 22522 -23460 | 312 | Ribonucleotide reductase,  R2/beta subunit (RNRR2)  (cd01049) | Ribonucleotide  reductase small  chain | 24R | 96 | 27R | 96 | 26R | 96 | RSIV 97 BAA82755 | | |
| 26L | 23812-24135 | 107 |  |  | 26L | 90 | 29L | 83 |  |  |  | | |
| 27L | 24157 -25053 | 298 | Xeroderma pigmentosum  G N- and I-regions  (XPGN, XPGI) (cd00128) | DNA repair protein RAD2 | 27L | 96 | 30L | 97 | 28L | 97 | RSIV 98 BAA82754  TFV 101R 34 AAL77816  FV3 95R 33 AAT09755  SGIV 97L 32 AAS18112  GIV66L 32 AAV91082  LCDV-1 32 NP_078767  LCDV-C169R 30 YP_073674  ATV 10L 34 YP_003781  CIV 369L 24 NP_149824  MIV 76L 27 YP_654648 | | |
| 28L | 25071-28577 | 1168 | RNA polymerase I subunit A N-terminus(smart00663); RNA polymerase II, subunit (KOG0260) | Largest subunit of the DNA- RNA polymerase | 28L | 96 | 31L | 97 | 29L | 97 | RSIV 97 BAA82753  GIV 71L 38 AAV91086  SGIV 104L 39 AAS18119  LCDV-C 191R 37 YP_025105  TFV 8R 40 NP_571990  FV3 8R 39 YP_031586  LCDV-1 16L 38 NP_078624  ATV 91R 39 YP_003777  MIV 90L 27 YP_654662 | | |
| 29L | 28584-28787 | 67 | C2C2 Zinc finger;  nucleic-acid-binding  motif in transcriptional  elongation factor TFIIS  and RNA polymerases  (smart00440); TFIIS,  transcription factor  S-II (TFIIS) (pfam01096) | Transcription elongation factor SII | 29L | 95 | 32L | 98 |  |  | SGIV 85R 38 AASQ18100  GIV 56R 26 AAV91074  FV3 81R 41 YP_031660  CIV 349L 28 NP_149812  TFV 86R 41 AAL77810  ATV 24L 41 YP_003795 | | |
| 30R | 29092 -29334 | 80 |  |  | 31R | 87 | 33R | 87 | 30R | 87 |  | | |
| 31R | 29464-30045 | 193 | Deoxynucleotide kinases  (COG1428) | Deoxyribonucle kinases | 32R | 92 | 34R | 87 | 31R | 87 | MIV 9R 28 YP_654601  TFV 91R 29 ABB92331  FV3 85R 29 YP_031664  LCDV-C 27R 26 YP_073536  LCDV-1 136R 26 NP_078725  GIV34 24 AAV91061  SGIV 67L 24 AAS18082  CIV 143R 27 NP_149606  ATV 19L 29 YP_003790 | | |
| 32L | 30030-30929 | 299 |  |  | 33L | 84 | 35L | 86 | 32L | 85 |  | | |
| 33R | 31000-34152 | 1050 | RNA polymerase  Rpb2, domain 6 25R  (pfam00562) | DNA-directed RNA polymerase II second largest subunit-like protein | 34R | 96 | 36R | 97 | 33R | 96 | LCDV-C25R 43 YP_073534  LCDV-1 25L 43 NP_078633  SGIV 73L 40 AAS18088  GIV 46L 40 AAV91067  TFV 65L 40 AAL77805  FV3 62L 39 YP_031641  MIV 9R 30 YP_654581  ATV 43R 39 YP_003815 | | |
| 34L | 34250-35362 | 370 |  |  | 35L | 82 | 37L | 85 | 34L | 85 | MIV9R 30 YP_654581 | | |
| 35R | 35442-36488 | 348 |  |  | 36R | 86 | 38R | 86 |  |  |  | | |
| 36L | 36515-37864 | 449 |  |  | 37L | 87 | 39L | 91 | 37L | 91 | LYCIV 90 ABI32393 | | |
| 37L | 37873-39306 | 477 |  |  | 38L | 83 | 40L | 87 | 38L | 87 |  | | |
| 38R | 39283-40218 | 311 |  |  | 39R | 86 | 41R | 87 |  |  |  | | |
| 39L | 40211-41359 | 382 |  |  | 40L | 84 | 42L | 85 | 41L | 86 |  | | |
| 40L | 41361-42689 | 442 |  |  | 41L | 95 | 43L | 89 | 42L | 89 |  | | |
| 41R | 42717-43310 | 197 |  |  | 42R | 84 | 44R | 90 |  |  |  | | |
| 42L | 43394-43756 | 120 | Erv1/Alt family  (pfam04777) | Thiol oxidoreductase | 43L | 99 | 45L | 99 |  |  | ATV 16L 41 AAP33193  LCDV-1 81R 39 NP_078699  LCDV-C 142L 38 YP_073647  FV3 88R 41 YP_031667  TFV 94R 40 AAB92342  SGIV 70R 37 AAS18085  GIV 43R 37 AAV91064  CIV 347L 29 NP_149810  MIV 96R 29 YP_654668 | | |
| 43L | 43759-44559 | 266 |  |  | 44L | 88 | 46L | 88 | 44L | 87 |  | | |
| 44L | 44565-45479 | 304 |  |  | 45L | 85 | 47L | 87 | 45L | 87 |  | | |
| 45L | 45473-46156 | 277 | Cytosine-C5-specific Cytosine DNA methylases  (cd00315) | Cytosine DNA Methylase transferase | 46L | 97 | 48L | 96 | 46L | 96 | ATV 21L 49 YP_003792  TFV 89R 48 AAL77813  FV3 83R 48 YP_031662  LCDV-1 5L 43 NP_078617  LCDV-C 86L 46 YP_025103 | | |
| 46R | 46316-46579 | 87 |  |  | 47R | 95 | 49R | 94 |  |  |  | | |
| 47R | 46576-46923 | 115 | Platelet-derived  and vascular  endothelial growth  factors (PDGF, VEGF)  family domain  (cd00135) | Vascular endothelial  growth factor like protein | 48R | 88 | 50R | 86 |  |  |  | | |
| 48L | 47169-47597 | 142 |  |  | 50L | 88 | 52L | 87 | 49L | 87 |  | | |
| 49L | 47825-48325 | 166 |  |  | 52L | 82 | 53L | 84 | 50L | 83 |  | | |
| 50R | 48327-48542 | 71 |  |  | 53R | 90 | 54R | 87 | 51R | 85 |  | | |
| 51L | 48555-49481 | 308 |  |  | 54L | 89 | 55L | 90 | 52L | 90 |  | | |
| 52L | 49504-50427 | 307 |  |  | 55L | 89 | 56L | 87 | 53L | 87 | LCDV-1 10L 50 NP_078687  FV3 19R 27 YP_031597  ATV 80L 23 AAP33261  SGIV 39L 24 AAS18054  GIV 21L 27 AAV91044  TFV 19R 24 ABB92284 | | |
| 53L | 50438-51085 | 215 |  | Helicase family | 56L | 98 | 57L | 98 | 54L | 98 | FV3 21L 42 YP_031599  ATV 78R 41 AAP33259  TFV 21L 41 ABB92286  SGIV 54R 39 AAS18069  LCDV-1 6L 44 NP_078618  LCDV-C 7L 37 YP_073516  CIV 67R 30 NP_149530  MIV 4R 33 YP_654576 | | |
| 54L | 51092-51352 | 86 |  |  | 57L | 96 | 58L | 94 | 55L | 96 |  | | |
| 55L | 51589-52104 | 171 |  |  |  |  | 59L | 86 | 56L | 86 |  | | |
| 56L | 52170-52973 | 267 |  | putative replication factor | 61L | 94 | 60L | 95 | 57L | 94 | TFV 105R 25 ABB92350  LCDV-C 75L 23 YP_073685  SGIV 116R 26 AAS18131  LCDV-1 162L 21 NP_078747  FV3 1R 24 YP_031579  ATV 91R 25 AAP33272  CIV 282R 29 NP_149745  GIV 79R 26 AAS18131 | | |
| 57L | 52970 -56419 | 1149 |  |  | 62L | 85 | 62L  61L | 80  94 | 58L | 84 |  | | |
| 58L | 56946-59594 | 882 | DEAD-like helicases  superfamily (cd00046) | SNF2 family helicase | 63L | 95 | 63L | 95 | 59L | 95 | SGIV 60R 32 AAS18075  LCDV-C 75L 32 YP_073582  LCDV-1 132L 31 NP_078720  GIV36R 32 AAV91057  FV3 9L 32 YP_031587  TFV 9L 32 AAL77795  ATV 7L 32 AAP33184  CIV 22L 27 NP_149485  MIV 87L 25 YP_654659 | | |
| 59L | 59637-61109 | 490 | Dual specificity  phosphatases (DSP);  Ser/Thr and Tyr  protein phosphatases  (cd00127) | mRNA capping  enzyme | 64L | 95 | 64L | 97 | 60L | 96 |  | | |
| 60L | 61151- 61630 | 159 | RING-finger  domain (cd00162) | RING-finger-containing E3 ubiquitin ligase | 65L | 88 | 65L | 88 | 61L | 88 |  | | |
| 61L | 61680- 62720 | 346 | RING-finger  domain (cd00162) | RING-finger-containing E3 ubiquitin ligase | 66L | 86 | 66L | 87 | 62L | 87 | RSIV 87 BAD98247 | | |
| 62L | 62948-63349 | 133 |  |  |  |  |  |  | 63L | 76 |  | | |
| 63L | 63544-64986 | 480 |  |  | 68L | 93 | 69L | 93 | 64L | 92 |  | | |
| 64L | 65044-65271 | 75 |  |  | 69L | 76 | 70L | 59 | 65L | 58 |  | | |
| 65L | 65858-67468 | 536 |  |  | 71L | 93 | 71L | 94 | 68L | 94 |  | | |
| 66R | 67509- 67940 | 143 |  |  | 73R | 82 | 72R | 79 |  |  |  | | |
| 67R | 67989-69011 | 340 |  |  | 74R | 92 | 73R | 95 | 70R | 95 |  | | |
| 68L | 69020- 69226 | 68 |  |  | 75L | 72 | 74L | 70 | 71L | 70 |  | | |
| 69R | 69284-72460 | 1058 |  |  | 76L | 94 | 75L | 95 | 72L | 91 | CIV 295L 31 NP_149758  FV3 41R 25 YP_031619  ATV 69R 25 AAP33249  LCDV-C 235R 24 YP_073738  SGIV 57L 23 AAS18072  LCDV-1 163R 22 NP_078748  TFV 45R 25 ABB92306  GIV 35L 23 AAV91056  MIV 16R 25 YP_654588 | | |
| 70R | 72116 -73609 | 497 | Ankyrin repeats  (cd00204) | Ankyrin repeats-containing | 77R | 92 | 76R | 92 |  |  |  | | |
| 71R | 73606-74070 | 154 |  |  | 78R | 94 | 77R | 92 | 75R | 92 |  | | |
| 72L | 74082-74465 | 127 |  |  | 79L | 78 | 78L | 86 | 76L | 86 |  | | |
| 73R | 74466-74843 | 125 |  |  |  |  | 79R | 81 | 77R | 81 | TV3 5R 27 YP_031583  TFV 6R 28 ABB392274 | | |
| 74R | 74850-75353 | 167 |  |  | 81R | 95 | 80R | 95 | 78R | 96 |  | | |
| 75L | 75394-76500 | 368 |  |  | 82L | 91 | 81L | 93 | 79L | 93 |  | | |
| 76R | 76549-76908 | 119 |  |  |  |  | 82R | 94 | 80R | 94 |  | | |
| 77L | 76942-78297 | 451 |  |  | 84L | 87 | 83L | 88 | 81L | 88 |  | | |
| 78R | 78324-78842 | 172 |  |  | 85R | 73 | 84R | 85 | 82R | 85 |  | | |
| 79R | 78839-79303 | 154 |  |  | 86L | 89 |  |  |  |  | LCDV-C 153L 50 YP_073658  TFV 100R 51 ABB92346  LCDV-1 19R 36 NP_078627  GIV 67R 42 AAV91083  FV3 94L 50 YP_031673  SGIV 98R 41 YP_164193  ATV 11L 50 YP_003782  MIV 33L 42 YP_654605  CIV 307L 41 NP_149770 | | |
| 80R | 79263-80066 | 267 | Ribonuclease  III family  (smart00535);  dsRNA-specific  ribonuclease  (COG0571) | Ribonuclease III | 87R | 95 | 85R | 97 | 83R | 97 | SGIV 84L 34 AAS18099  GIV 26L 33 AAV91073  LCDV-C 187R 31 YP_073691  LCDV-1 137R 29 NP_078726  FV3 80L 28 YP_031659  TFV 85L 28 NP_572005  ATV 25R 28 AAP33202  CIV 142R 30 NP_149605  MIV 101R 23 NP_149605 | | |
| 81R | 80256- 80573 | 105 |  |  | 88R | 97 | 86R | 92 | 84R | 97 |  | | |
| 82R | 80528-82096 | 522 |  |  | 88R | 91 | 87R | 94 | 84R | 93 |  | | |
| 83L | 82077- 83093 | 338 |  |  |  |  |  |  | 85L | 93 | GIV 4R 40 AAV91031  SGIV19R 40 YP_164114  FV3 2L 38 YP_031580  ATV 1L 37 YP_003772  LCDV-C 38R 36 YP_073546  LCDV-1 160L 35 NP_078745  MIV 47R 34 YP_654619  TFV 2L 35 ABB92270  CIV 337L 30 NP_149800 | | |
| 84R | 83094-83273 | 59 |  |  | 92R | 92 | 89R | 91 |  |  |  | | |
| 85L | 83270-84196 | 308 |  |  | 93L | 92 | 90L | 94 | 86L | 93 |  | | |
| 86L | 84206-84703 | 166 |  |  | 94L | 93 | 91L | 92 | 87L | 92 |  | | |
| 87L | 84724-85893 | 389 |  |  |  |  | 92L | 91 | 88L | 91 |  | | |
| 88L | 85901- 86653 | 250 |  |  | 96L | 97 | 93L | 97 |  |  | LCDV-1 108L 25 NP_078701  LCDV-C 100L 30 YP_073606  ATV 87R 28 NP_003862  TFV 12L 27 ABB92278  FV3 12L 27 AAT09671  GIV 80R 26 AAV91095  SGIV 118R 31 AAS18133  MIV 287R 20 NP_149750  CIV 287L 20 NP_149750 | | |
| 89L | 86644-87129 | 161 |  |  |  |  | 94L | 93 | 90L | 93 |  | | |
| 90L | 87177-87533 | 118 | RING-finger  domain (cd00162) | RING-finger  domain-containing E3 protein | 99L | 76 | 95L | 80 | 91L | 80 |  | | |
| 91L | 87551-88129 | 192 |  |  | 100L | 81 | 96L | 84 | 92L | 85 |  | | |
| 92L | 88175-88690 | 171 |  |  | 101L | 93 | 97L | 94 |  |  |  | | |
| 93R | 88754-90184 | 476 | Ankyrin repeats  (cd00204) | Ankyrin repeat containing  protein | 102R | 88 | 98R | 91 | 94R | 91 |  | | |
| 94R | 90187-90573 | 128 | Src homology 2  domains (cd00173) | Suppressor of  cytokine signaling  protein | 103R | 77 | 99R | 75 |  |  |  | | |
| 95R | 90608- 91384 | 258 |  |  | 104R | 85 | 100R | 84 | 96R | 84 |  | | |
| 96R | 91386-91757 | 123 |  |  | 105R | 91 |  |  | 97R | 95 |  | | |
| 97L | 91882- 92913 | 343 |  |  | 106L | 84 | 103L | 75 | 99L | 73 |  | | |
| 98L | 93050-93265 | 71 |  |  | 107L | 88 | 104L | 93 |  |  |  | | |
| 99L | 93639-96401 | 920 | Predicted ATPase  (COG3378); Poxvirus  D5 protein-like  (pfam03288) | D5 family NTPase | 109L | 97 | 106L | 98 | 101L | 98 | LCDV-C 80L 35 YP_073585  SGIV 52L 36 AAS18067  GIV 31L 36 AAV91054  FV3 22R 35 YP_031600  ATV 77L 35 AAP33258  LCDV-1 128L 34 NP_078717  TFV 22R 35 ABB92287  MIV 121R 27 YP_654693  CIV 184R 26 NP_149647 | | |
| 100R | 96410-96604 | 64 |  |  | 110R | 94 | 107R | 92 |  |  |  | | |
| 101L | 96601-97497 | 298 | Tnf receptor-associated  factor 2 domain  (cd00270); | Tumor necrosis  factor type 2  receptor associated  protein | 111L | 89 | 108L | 87 | 102L | 90 | RSIV 89 BAD98248 | | |
| 102R | 97518- 98261 | 247 |  | Proliferating cell  nuclear antigen | 112R | 97 | 109R | 98 | 103R | 99 | LCDV-1 3L 31 NP_078615  GIV 41L 28 AAV91062  LCDV-C 197L 29 YP_073700  SGIV 68L 28 AAS18083  ATV 20L 24 AAP33197  FV3 84R 25 YP_031663  TFV 90R 25 ABB92338 | | |
| 103R | 98369-98521 | 50 |  |  | 113R | 100 | 110R | 94 |  |  |  | | |
| 104R | 98534:98749 | 71 |  |  | 113R | 100 | 110R | 95 |  |  |  | | |
| 105L | 98793-101375 | 860 | CAP10, putative  lipopolysaccharide- modifying enzyme  (smart00672) | Tyrosine kinase | 114L | 94 | 111L | 94 | 106L | 91 | FV3 27R 44 YP_031605  TFV 29R 44 AAL7779  ATV 58R 29 AAP33237  SGIV 81L 39 AAS18093  LCDV-C 173R 23 YP_073677  CIV 179R 21 NP_149902  LCDV-1 195R 20 NP_078770  MIV 035R 22 YP_654607  GIV 52L 37 AAV91071 | | |
| 106R | 101972-102982 | 336 |  | Immediate early  protein ICP-46 | 115R | 96 | 112R | 94 | 108R | 87 | LCDV-1 47L 23 NP_078648.1  LCDV-C 162R 25 YP_073667  TFV 97R 24 NP_572011  FV3 91R 24 YP_031670  MIV 039R 27 YP_654611  ATV 13L 24 AAP33190.1  SGIV 162L 20 AAS18177  CIV 393L 20 NP_149856  GIV 108L 20 AAV91117 | | |
| 107R | 102801-104411 | 536 |  |  | 116R | 87 | 113R | 82 | 110R | 89 | Sea perch iridovirus 46 AAV51312  OSGIV 25L 46  RBIV 22L 46  RSIV 45 AAQ07955  DGIV 45 AAY58051 | | |
| 107R | 103038-104411 | 457 |  |  | 116R | 87 | 113R | 82 | 110R | 91 | Sea perch iridovirus 46 AAV51312  OSGIV 25L 46  RBIV 22L 46  RSIV 45 AAQ07955  DGIV 45 AAY58051 | | |
| 108L | 104463-105137 | 224 |  | Early 31 kDa  protein | 117L | 94 | 114L | 95 | 111L | 96 | FV3 23R 25 YP_031603  TFV 25R 25 AAL77797  LCDV-C 34L 23 YP_025101  LCDV-1 122R 24 NP_078713  ATV 55R 23 AAP33234  SGIV 6R 22 AAS18021  GIV 96 23 AAV91123 | | |
| 109L | 105473-106783 | 436 | Ankyrin repeat  （cd00204） | Ankyrin repeat-containing | 118L | 92 | 115L | 91 | 112L | 93 |  | | |
| 110R | 106777-107133 | 118 | RING-finger  domain (cd00162) | RING-finger  domain-containing E3 protein | 119R | 96 | 116R | 93 | 113R | 93 |  | | |
| 111R | 107160 -107666 | 168 |  |  | 120R | 89 | 117R | 89 | 114R | 78 |  | | |
| 112L | 107667 -108317 | 216 |  |  | 121L | 70 | 118L | 75 | 115L | 69 |  | | |
| 113R | 108327-109046 | 239 |  | ATPase | 122R | 98 | 119R | 98 | 116R | 98 | GSIV 98 AAL68653  LBIV 98 AAL68654  RSIV 98 BAA28670  SBIV 98 BAA96406  GIV 98 BAA96407  LYCIV 98 ABI32392  DGIV 98 AAP74205  ALIV 98 BAA96408  Olive flounder iridovirus 99 ABY16713  LCDV-C 114L 54 YP_073620  LCDV-1 54R 54 NP_078656  SGIV 134L 52 YP_164229  GIV90L 52 AAV91100  FV3 15R 51 YP_031593  ATV 83L 51 YP_003858  TFV 16R 51 AAL77796  CIV 75L 39 NP_149538  MIV 88L 37 YP_654660 | | |
| 114R | 109124-109318 | 64 |  |  | 123R | 92 | 120R | 93 | 117R | 94 |  | | |
| 115L | 109407-110093 | 228 | Ankyrin repeat  （cd00204） | Ankyrin repeat-containing | 124L | 92 | 121R | 93 | 118L | 84 |  | | |

aa: number of amino acids of each TRBIV putative protein.

*: best matched ORF from the ISKNV, OSGIV, and RBIV genome.
